# Short-Term Exposure to Tebuconazole Triggers Haematological, Histological and Biochemical Disturbances in Rainbow Trout (*Oncorhynchus mykiss*)

**DOI:** 10.3390/toxics13080630

**Published:** 2025-07-27

**Authors:** Akif Er

**Affiliations:** Department of Aquaculture, Faculty of Fisheries, Recep Tayyip Erdoğan University, 53100 Rize, Türkiye; akif.er@erdogan.edu.tr; Tel.: +90-54-3952-4471

**Keywords:** tebuconazole (TBZ), rainbow trout, acute toxicity, haematological parameters

## Abstract

Tebuconazole (TBZ), a triazole-class fungicide widely used in agriculture, is frequently detected in aquatic environments due to runoff and leaching, where it poses a threat to non-target aquatic organisms. This study investigates the acute toxicity of TBZ on juvenile rainbow trout (*Oncorhynchus mykiss*), a commercially important cold-water fish species. The 96 h LC_50_ value was determined to be 9.05 mg/L using probit analysis. In addition to mortality, the physiological responses of fish exposed to both LC_50_ and maximum tolerance concentration (MTC; 6 mg/L) were evaluated through haematological and histological assessments. TBZ exposure significantly suppressed key haematological parameters, particularly WBC, RBC, HGB, HCT, and LYM, indicating immunosuppression and potential hypoxia. Histological examination revealed progressive and regressive damage in gill tissues, including epithelial lifting, hyperplasia, and hypertrophy, which were more severe in the LC_50_ group. These alterations were quantified using a semi-quantitative scoring system. Additionally, significant changes in biochemical parameters such as ALT, AST, creatinine, total protein, and glucose levels were observed, further indicating hepatic and renal dysfunctions induced by TBZ exposure. The findings demonstrate that TBZ exposure induces substantial physiological and structural impairments in rainbow trout, highlighting the importance of assessing the ecological risks of fungicide contamination in aquatic environments. The study also provides a dose–response model that can be used to estimate mortality risk in aquaculture operations exposed to TBZ.

## 1. Introduction

The increasing global demand for food, driven by the rapid growth of the world population, has led to a continuous rise in the need for agricultural products. Consequently, substantial amounts of pesticides are reportedly used to protect crops against harmful organisms [1,2]. These pesticides eventually enter aquatic ecosystems through surface runoff, rainfall, infiltration, and atmospheric deposition [3]. Once in these environments, they exert toxic effects on non-target organisms [4]. It is estimated that less than 0.3% of applied pesticides reach their intended targets, while the remaining 99.7% contribute to environmental contamination and pose threats to public health [5]. Therefore, this heavy reliance on pesticides in agricultural production has become a major environmental concern, and pesticides are now considered among the priority pollutants in receiving environments [6,7]. Currently, the registration and approval processes of pesticides are generally based on their individual toxicity data [8].

Triazole group fungicides are widely used in agriculture to control pathogenic fungi and to prevent the development of resistance [9]. These fungicides act by specifically inhibiting the ergosterol biosynthesis pathway in plants, a mechanism crucial for the integrity of fungal cell membranes [10]. Tebuconazole (TBZ) is a prominent triazole fungicide commonly used in agricultural production to manage fungal diseases [11]. It functions as a CYP450 inhibitor, targeting the fungal enzyme CYP51, which catalyses the sterol 14α-demethylation reaction, and it exhibits high affinity for fungal cytochrome P450 enzymes [12]. As a result, TBZ has become one of the most extensively sold fungicides globally, with an estimated usage of 2.5 million pounds reported in 2016 alone [13]. Although TBZ is generally classified as a fungicide with low acute toxicity, it demonstrates high persistence in aquatic environments, with a reported half-life of approximately 600 days [14]. Accordingly, concentrations of TBZ in surface waters have been reported to range from 0.6 to 200 μg/L [15,16]. Due to its environmental stability and tendency to accumulate, TBZ is frequently detected in aquatic ecosystems. This persistence raises concerns about its potential to exert cumulative effects on aquatic organisms and humans through mixture toxicity [17].

As a fungicide belonging to the azole group, TBZ can be transported into aquatic environments through agricultural practices, leading to various physiological and biochemical disturbances in fish. It has been demonstrated that TBZ induces oxidative stress by triggering the production of reactive oxygen species (ROS), resulting in lipid peroxidation, protein carbonylation, and the disruption of antioxidant defence mechanisms such as SOD, CAT, and GST [18,19,20]. These effects of TBZ are further supported by metabolic changes observed in species like zebrafish (*Danio rerio*) and common carp (*Cyprinus carpio*), including glycogen depletion, glucose imbalance, increased lactate levels, and reductions in protein concentrations [21,22]. Additionally, even short-term exposure to sub-lethal doses has been reported to cause severe effects such as irregularities in liver morphology, glycogen depletion, hepatocyte shrinkage, and alterations in superoxide dismutase levels [23].

TBZ’s tendency to bioaccumulate and its persistence in tissues contribute to the prolonged manifestation of its ecotoxicological effects [15,16]. In this context, it should be carefully assessed in terms of environmental risk, as it has been shown to exhibit a half-life of 24 days and a complete elimination time of 105 days in zebrafish tissues [24]. The observed increase in Na^+^/K^+^-ATPase activity indicates that TBZ also affects ion balance and osmoregulatory processes [25]. Furthermore, this compound causes endocrine disruption at the hormonal level, leading to increased vitellogenin levels and imbalances in triglyceride and lactate concentrations, thereby disturbing metabolic homeostasis [26]. The use of TBZ in combination with other compounds may further enhance its toxicity; for example, co-exposure with difenoconazole has been shown to induce transcriptomic and metabolomic alterations in liver and gonadal tissues [9]. In conclusion, TBZ poses a substantial threat to fish health not only due to its acute toxicity but also because of its chronic effects, bioaccumulation potential, and capacity to cause metabolic disturbances. However, in order to understand the chronic effects and sub-lethal bioaccumulation of this fungicide, it is essential to first conduct acute toxicity studies and monitor the associated physiological responses.

The potential effects of TBZ used in agriculture on non-target fish species may, in turn, jeopardise other agricultural activities, such as aquaculture. In this context, the adverse impacts of this chemical on commercially important fish species have become an important area of research. Rainbow trout (*Oncorhynchus mykiss*) is the most widely farmed cold-water fish species worldwide and holds significant market value [27]. Due to its position at a high trophic level, it is highly sensitive to environmental conditions, including water quality parameters and the presence of aquatic pollutants [28,29,30]. Therefore, rainbow trout may be exposed to TBZ both in aquaculture systems and in the wild. The objective of the present study is to determine the 96 h lethal concentration (LC_50_) of TBZ in rainbow trout reared under culture conditions, and to investigate the physiological responses exhibited by the fish upon exposure to TBZ.

## 2. Materials and Methods

### 2.1. Experimental Organism, Toxic Material and Water Quality Parameters

The rainbow trout (*Oncorhynchus mykiss*) used in this study were obtained from a private aquaculture facility located in Rize, Türkiye. The acute toxicity experiment was designed using ten fish per treatment group, with an average body weight of 95.4 ± 4.2 g. Upon arrival, the fish were acclimated to the laboratory conditions and water for two weeks. To eliminate the effects of recent feeding on physiological parameters, the fish were fasted for two days prior to the start of the experiment and were not fed during the exposure period. All experimental procedures were reviewed and approved by the Local Ethics Committee of Recep Tayyip Erdoğan University (Decision No: 2023/08).

The toxicant used in the experiment, tebuconazole (TBZ) (25% purity, Bayer, FOLICUR^®^ WP 25, produced in Türkiye), was purchased commercially. TBZ is a group G1:3 fungicide sold in solid formulation. In accordance with the experimental design, the fungicide was weighed using a precision balance, and target concentrations were prepared accordingly. Since the substance is water-soluble, no organic solvents were required.

Water quality parameters—including dissolved oxygen (DO; mg/L), temperature (T; °C), electrical conductivity (EC; mS/cm), and pH—were routinely monitored using a portable multi-parameter device (Hach, HQ40D 58258-00, Loveland, CO, USA). The applied fungicide did not alter water quality parameters. Recorded values during the experiment were as follows: DO: 9.2 ± 1.7 mg/L, T: 18.12 ± 0.34 °C, EC: 5.1 ± 0.1 mS/cm, and pH: 7.1 ± 0.5. These parameters remained within acceptable limits to sustain the acute phase requirements for the experimental fish species used.

### 2.2. Experimental Design of Acute Toxicity Study

The acute toxicity experiment was conducted at the Application and Research Center of the Faculty of Fisheries, Recep Tayyip Erdoğan University. All tanks used in the study had a water volume of 70 L, and the experiment was carried out under a controlled photoperiod of 12 h light and 12 h dark. The water used in the system was groundwater with a salinity of 3‰. A centralised blower system was employed to provide aeration to all tanks.

The lethal concentration during the acute exposure period is defined as the concentration of a substance that results in 50% mortality of the target organism population at specific time points: 24th, 48th, 72nd, and 96th h [31]. The acute trial was designed using six different concentration groups of TBZ: 7.5 mg/L, 9.0 mg/L, 10.5 mg/L, 12.0 mg/L, 15.0 mg/L, and 18.0 mg/L. Tanks were established in triplicates, with ten fish in each tank and a total of thirty fish per treatment group. The control group did not receive any TBZ exposure. For each time point, LC_10_, LC_50_ and LC_90_ values were calculated and are presented in Table 1. The relationship between mortality rate and the logarithmic concentrations of TBZ was modelled using a four-parameter logistic regression equation. Curve fitting was performed using the Nonlinear Curve Fit tool in OriginPro 2022 (OriginLab Corporation, Northampton, MA, USA). The model used the equation

$$y=A1+(A2-A1)/(1+10^{\log_{x}0-x}\times p),$$

where *y* is the mortality rate, *A*1 and *A*2 are the bottom and top asymptotes, respectively, *x* is the log-transformed concentration of TBZ, and *p* is the Hill slope. All parameters were estimated using the least squares method. Based on results, the 96 h LC_50_ value (representing the worst-case scenario: 9 mg/L) was selected for evaluating haematological and histological responses in fish. Prior to the main experiment, preliminary range-finding tests were conducted to determine the Maximum Tolerated Concentration (MTC) of tebuconazole (TBZ) for rainbow trout. In these trials, fish were exposed to a series of increasing TBZ concentrations (ranging from 1 to 10 mg/L) over a 96 h period. The purpose of these trials was to identify the highest concentration that resulted in no mortality within this exposure duration. Based on the results, 6 mg/L was established as the highest concentration at which 100% survival was observed throughout the 96 h test period (Table 1). This concentration was therefore designated as the MTC and used for subsequent haematological and histopathological evaluations in the main study.

### 2.3. Haematological Examination

At the end of the acute exposure period, five fish from each tank in the LC_50_ and MTC groups were randomly selected for haematological analysis. The fish were first anaesthetised using clove oil at a concentration of 60 mg/L. Blood samples (1 mL) were then collected from the caudal vein using 2.5 mL syringes. Immediately after collection, the blood was transferred into K_3_ EDTA tubes for analysis. The following haematological parameters were measured: leukocyte count (WBC), erythrocyte count (RBC), haemoglobin concentration (HGB), haematocrit value (HCT), lymphocyte count (LYM), mean corpuscular volume (MCV), mean corpuscular haemoglobin (MCH), and mean corpuscular haemoglobin concentration (MCHC). All haematological parameters were analysed using an automated haematology analyser (Prokan Pe-6800 Vet, Wuhan Aliroad Medical Equipment Co., Ltd., Shenzhen, China). Prior to analysis, the device was calibrated and quality-checked specifically for fish blood samples [32].

### 2.4. Histological Evaluation

Three fish were randomly selected from the LC_50_ and MTC group tanks for histological evaluation. For this purpose, the fish were thoroughly anaesthetised using clove oil at a concentration of 100 mg/L. For gill tissue sampling, the second lamella of the second gill arch was excised. The collected tissue samples were fixed in 10% neutral buffered formalin. After 24 h, samples were transferred to 50% ethanol for preservation. During the histological preparation process, the tissues were rinsed in running water to remove residual alcohol. Subsequently, the tissues were processed through a series of alcohol and xylene baths, and then embedded in paraffin at +65 °C overnight. In the next step, paraffin-embedded gill tissues were sectioned into 5 μm thick slices using a microtome and mounted onto glass slides. The slides were incubated at +65 °C to remove paraffin. Following paraffin removal, the tissues were treated with xylene and then stained using hematoxylin and eosin (H&E) protocols. Finally, the slides were mounted with entellan and covered with coverslips. The prepared histological samples were examined under a light microscope, and pathological findings were documented photographically [33].

To quantify the histological findings, a semi-quantitative model was used [34]. According to this model, histopathological alterations are categorised into five reaction patterns: circulatory disturbances, regressive changes, progressive changes, inflammation, and tumours. However, since some of these patterns were not observed in the present study, only two patterns were evaluated. These reaction patterns were determined based on the observed pathological changes in the organs. In the histological assessment tool, the importance factor represents the pathological relevance of each lesion on a scale of 1 (minimal) to 3 (marked). The score value for each histological alteration reflects its severity and is quantified using a Likert scale ranging from 0 to 6 (Table 2) [35]. The final score for each lesion was calculated by multiplying its importance factor by the respective score value. For each reaction pattern, the total score was obtained by summing the individual lesion scores.

### 2.5. Biochemical Analysis

At the end of the 96 h acute period, six fish were randomly selected from the control, MTC, and LC50 groups. Blood samples were collected from the caudal vein of the fish using 2 mL syringes and transferred to heparinized tubes. The samples were centrifuged at 5000 rpm for 7 min, and the supernatant plasma layer was carefully separated and stored at −80 °C until analysis. Biochemical parameters were determined as alanine aminotransferase (ALT), aspartate aminotransferase (AST), alkaline phosphatase (ALP), urea, and glucose levels. Analyses were performed using a Randox LT406-RX (Randox Laboratories Ltd., Crumlin, UK) fully automated, random-access clinical biochemistry analyser [36].

### 2.6. Statistical Analysis

All data are presented as the means ± standard deviation (SD). Whether all datasets had a normal distribution was tested using the Kolmogorov–Smirnov test. Accordingly, since the datasets showed a normal distribution, a one-way ANOVA test was performed. In cases where significant differences were found between groups, Tukey’s test was conducted to evaluate these differences. To determine the LC_50_ value, a Probit test was applied. *p*-values less than 0.05 were considered statistically significant. All datasets were analysed using the SPSS 25 software package for Windows (Version 25, IBM Corp., Armonk, NY, USA). In addition, a dose–response graph was created based on the Log_10_ TBZ concentration using OriginLab 2025 Student Version.

## 3. Results

### 3.1. Acute Toxicity of Pesticides for Rainbow Trout

**Figure 1:**
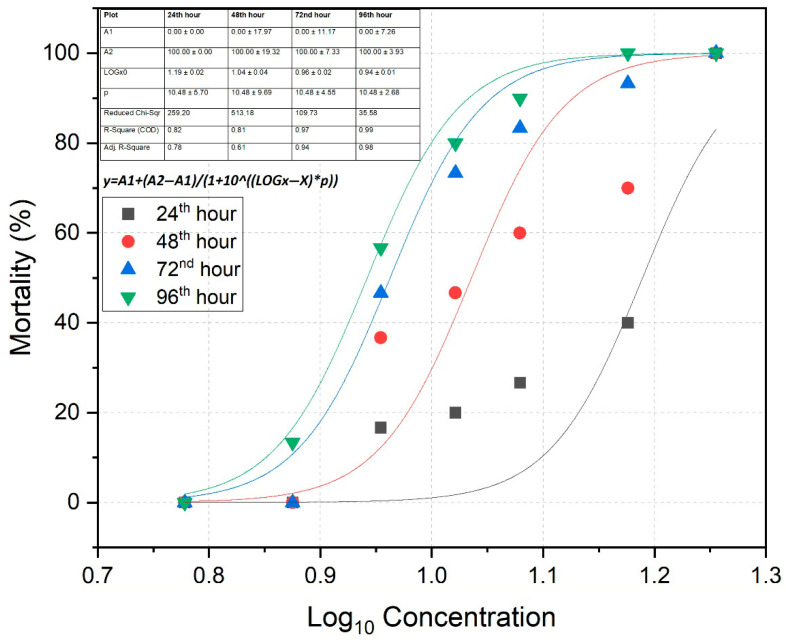
Dose–response curve (Log_10_-transformed) of rainbow trout following exposure to varying concentrations of tebuconazole.

The mortality of fish in response to the logarithmic (Log_10_) concentrations of TBZ during the acute exposure period is presented in Figure 1. Based on Probit analysis, the LC_50_ value at the 96th hour was calculated as 9.05 mg/L, with a 95% confidence interval. The mortality rate equation corresponding to TBZ concentration at each time interval (24th, 48th, 72nd, and 96th h) is as follows: $y=A1+(A2-A1)/(1+10^{\log_{x}0-x}\times p)$. In this equation; *y*: mortality rate, *A1*: bottom asymptote, *A2*: top asymptote, *x*: TBZ concentration; and *p*: hill slope. All model parameters for each time point are presented in the table within Figure 1. No mortality was observed in the control group and in tanks containing 6 mg/L TBZ throughout the 96 h exposure period.

### 3.2. Haematological Parameters

At the end of the experiment, various haematological parameters for the LC_50_, MTC, and control groups are presented in Table 3. The WBC count in the control group was significantly higher than in the other two treatment groups (*p* < 0.01). Both RBC and HGB levels in the control group were significantly higher compared to the LC_50_ group (*p* < 0.01). For HCT and MCV, the values in the LC_50_ group were observed to be significantly lower than those in the other groups (*p* < 0.05). The LYM values showed statistically significant differences among all groups, following the pattern control > MTC > LC_50_ (*p* < 0.01). No statistically significant differences were found between groups for MCH and MCHC (*p* > 0.05).

### 3.3. Histological Alterations

**Figure 2:**
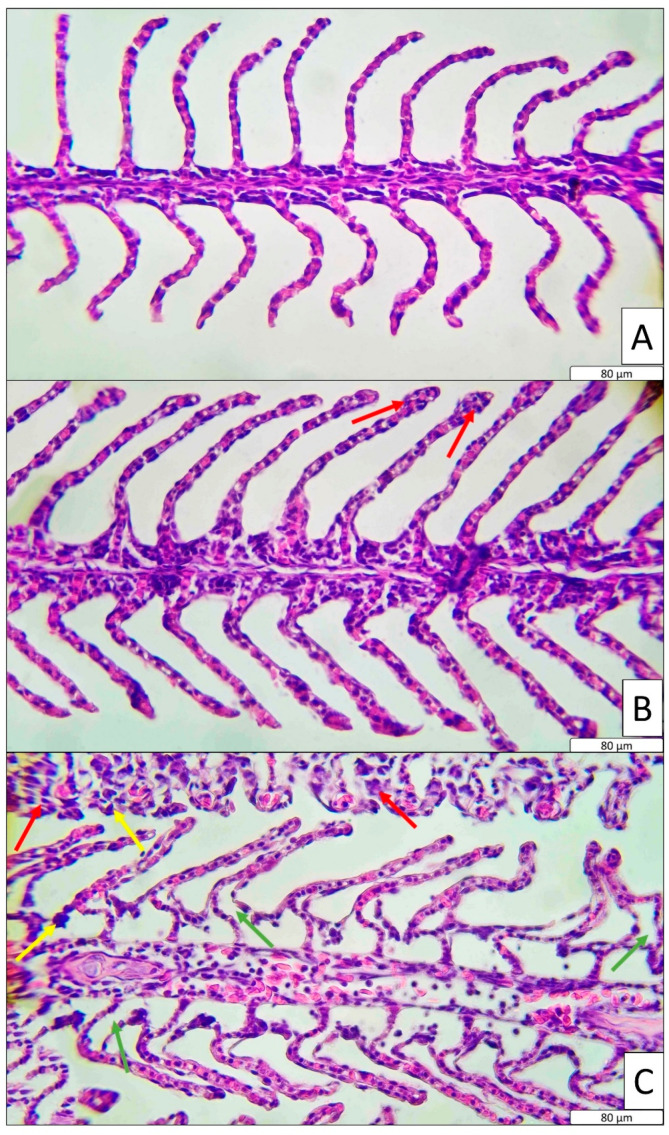
Histological observation of gill in control, MTC and LC_50_ groups. (**A**): Control, (**B**): MTC and (**C**): LC_50_. Histological symptoms are shown with arrows (red: hyperplasia, yellow: hypertrophy, green: epithelial lifting).

Gill tissues were histologically evaluated in the control, MTC, and LC_50_ groups (Figure 2). The gill tissue in the control group represented a healthy and intact gill structure. In contrast, moderate hyperplasia and epithelial lifting were observed in the gill tissues of the MTC group, along with sporadic mild hypertrophic cells (Table 4). Furthermore, the gill tissues of fish in the LC_50_ group exhibited very severe epithelial lifting and hyperplasia (Table 4). Compared to the other two groups, hypertrophy symptoms were also more pronounced and severe in this group.

According to the semi-quantitative histological model (Table 5), the LC_50_ group presented significantly higher scores for both regressive and progressive changes, whereas the control group showed significantly lower values (*p* < 0.01). The calculated organ index values for the control, MTC, and LC_50_ groups were 4.70, 8.82, and 20.76, respectively.

### 3.4. Biochemical Results

**Figure 3:**
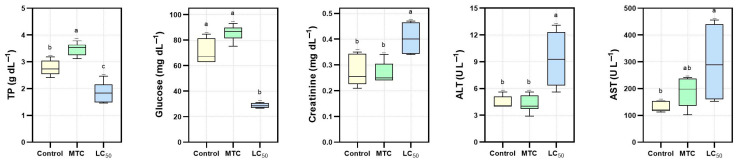
Plasma biochemical parameters measured in Control, MTC, and LC_50_ groups. Different letters (a, b, c) above the bars denote statistically significant differences among groups (*p* < 0.01).

Biochemical tests were performed on blood plasma samples of control, MTC and LC50 groups (Figure 3). Creatinine, ALT and AST values were found to be significantly higher in the LC50 group than in the control group (*p* < 0.01). The MTC group generally exhibited similar results to the control group. For TP and glucose, the LC50 group was significantly lower than the control and MTC groups (*p* < 0.01).

## 4. Discussion

Aquatic environments are the ultimate recipients of anthropogenic pollutants, including pesticides used in agriculture [37]. Among these contaminants, the toxic effects of pesticides on organisms have been previously documented [38]. In this context, TBZ, a widely used fungicide, has been reported to exhibit developmental toxicity, thyroid toxicity, and reproductive toxicity in various organisms. It has also been shown to induce endocrine disruption in male zebrafish and reduce reproductive output in Daphnia magna [39,40]. In the present study, the 96 h LC_50_ value for juvenile rainbow trout exposed to TBZ was determined to be 9.05 mg/L. Previous studies have reported lower LC_50_ values for this pesticide in other species: 2.37 mg/L for common carp (*Cyprinus carpio*) [41], 1.13 mg/L for *Chelon auratus* [42] and 7.2 mg/L for the local species *Cirrhinus mrigala* [14]. For zebrafish (*Danio rerio*), similar LC_50_ values have been reported in two different studies as 8.73 mg/L and 8.16 mg/L, respectively [17,43]. In contrast, a higher LC_50_ value of 19.63 mg/L was previously reported for the same species [26]. While these studies demonstrate the effects of TBZ on warm freshwater fish and model organisms, the current study addresses a gap in the literature by providing data on a commercially valuable cold water species such as rainbow trout. One of the novel contributions of this study is the development of a mortality prediction model for rainbow trout exposed to TBZ, represented by the equation [$y=A1+(A2-A1)/(1+10^{\log_{x}0-x}\times p)$]. Using this model, the expected mortality rate in a fish population exposed to a known TBZ concentration can be predicted. This enables both small- and large-scale aquaculture facilities to anticipate potential risks and implement appropriate protective measures in advance. The model was derived from a dose–response curve generated through global fit in a multi-data fit mode and was previously applied in another study conducted by our group [44].

Although the TBZ concentrations in this study were above levels typically reported in natural surface waters [45,46,47,48], this is justified from a prospective toxicological perspective. Factors such as increasing agricultural intensification, expanding urbanisation, and the ineffectiveness of conventional wastewater treatment plants may lead to increased TBZ levels in freshwater systems in the future. In this context, assessing the acute toxic effects of high TBZ concentrations on aquatic organisms such as rainbow trout serves two important purposes: (i) contributing to the understanding of the consequences of exposure under worst-case scenarios and (ii) providing the basis for proactive management of environmental risks. Furthermore, such studies raise public awareness and provide decision-makers at the local or national level with a scientific basis for establishing water quality standards and regulating pesticide use. Similarly, predictive toxicological approaches have been adopted in various studies on the potential pollutants and their ecological impacts under future land use and climate change scenarios [49,50].

Haematological parameters are among the fastest and most sensitive indicators of stress in organisms exposed to toxic substances [51]. In the present study, overall blood parameters were significantly lower in the LC_50_ group. In the MTC group, lower values of WBC and LYM compared to the control group were particularly notable. The significant reductions observed in the LC_50_ group can be interpreted as follows: The decline in WBC, the first line of defence in the immune system, indicates an immunosuppressive effect of TBZ [52]. At high levels of toxicity, immune suppression may occur [35]. Additionally, the reduction in LYM may reflect TBZ’s suppressive impact on lymphoid tissues, suggesting potential immunological damage. The observed decrease in RBC could be indicative of TBZ’s toxicity to hematopoietic tissues [53,54], which would impair the oxygen-carrying capacity of the blood and consequently reduce the fish’s survival potential [55]. This is further supported by the decline in HGB, which may also result from haemolysis induced by TBZ-related stress [56,57]. The reduction in HCT reflects a decreased total erythrocyte volume in circulation, another indicator of TBZ-induced haematological toxicity. The decline in erythrocyte volume suggests microcytic anaemia, possibly indicating that TBZ disrupts intracellular water balance or the maturation process of erythrocytes [58]. Similar haematological effects of TBZ exposure have previously been reported in *Alburnus tarichi* [12].

Tracking histopathological alterations in organisms following exposure to toxic substances serves as a reliable indicator of the severity of the toxic effect [59]. In this context, the first tissues to be affected in fish exposed to toxicants are typically the gill tissues, due to their continuous contact with the aquatic environment [60]. In the present study, fish in the LC_50_ group exposed to TBZ exhibited severe hyperplasia and epithelial lifting, as well as moderately pronounced hypertrophy. A similar but less severe pattern was also observed in the MTC group. Gills are the primary organs for gas exchange in fish [61]. Structural damage such as hyperplasia (abnormal proliferation of epithelial cells), hypertrophy (enlargement of cells) and epithelial lifting (detachment of epithelium from lamellae), as observed in the LC_50_ group, significantly reduce the gill surface area and impair oxygen diffusion [62]. Consequently, this reduction in oxygen transfer leads to hypoxia, which may explain the suppressed hematopoietic activity or RBC destruction observed in exposed fish. Moreover, inadequate oxygen uptake via the gills can result in insufficient oxygen delivery to tissues, leading to oxidative degradation of erythrocytes [63]. Additionally, the direct toxic effect of TBZ on the gill epithelium may trigger systemic haematological responses via local inflammation and cytokine release. Notably, epithelial lifting and hypertrophy can facilitate the entry of foreign agents, increasing the risk of secondary infections; however, the immunosuppressive effects of TBZ may compromise immune cell activity. Similar epithelial alterations and lamellar deformations have been reported in Mediterranean mussels exposed to TBZ [64]. This study also provides semi-quantitative evidence that the “cellular and morphological changes” reaction pattern was significantly more pronounced in TBZ-exposed groups. According to the same model, both regressive and progressive changes were more severe in the TBZ-exposed groups of the present study. Comparable findings have also been reported in another quantitative model evaluating liver histology, where higher adverse Mean Assessment Values were observed in TBZ-treated groups [23]. In our previous study, we also employed the semi-quantitative model developed by Bernet et al., 1999 [34], and observed significantly higher scores for regressive changes, progressive changes and circulatory disturbances in Danube sturgeon exposed to malathion [44].

Biochemical parameters evaluated in the study showed significant changes in rainbow trout exposed to TBZ. Total protein (TP) levels increased significantly in the MTC group and decreased significantly in the LC_50_ group compared to the control group. This suggests that TBZ may suppress protein synthesis at high concentrations and negatively affect liver function [41]. Glucose levels remained elevated in the control and MTC groups but decreased significantly in the LC_50_ group. This suggests that high-dose TBZ exposure may suppress energy metabolism and induce a hypoglycemic response [65,66]. Creatinine levels increased significantly with increasing TBZ concentration, which can be interpreted as an indicator of deterioration in renal function and renal stress [67]. The significant increases in ALT and AST levels, particularly in the LC_50_ group, suggest that TBZ has hepatotoxic effects and may cause liver damage. This has also been reported in previous studies [12]. These biochemical changes indicate that TBZ may produce significant toxic effects not only for the haematological system but also on vital organs such as the liver and kidney.

## 5. Conclusions

The present study investigates the toxic effects of TBZ, a commonly used agricultural fungicide and anthropogenic pollutant, on rainbow trout. Specifically, the 96 h LC_50_ value of TBZ for rainbow trout was determined, and the fish’s physiological responses to both this lethal concentration and the highest non-lethal concentration (MTC) were evaluated. Exposure to TBZ altered haematological parameters, negatively impacting the immune system of the fish and inducing hypoxic conditions by impairing oxygen transport. Histological findings further confirmed the adverse effects of TBZ, revealing marked structural damage in gill tissues. These histopathological changes are presumed to be a major factor contributing to the haematological disruptions, and were validated using a semi-quantitative assessment model. In addition, significant alterations in plasma biochemical parameters—particularly ALT, AST, creatinine, glucose, and total protein—indicate hepatic and renal dysfunctions, further supporting the systemic toxicity of TBZ. The results of this study highlight the potential risk that pesticide use poses to non-target aquatic organisms and offer critical insight for decision-makers. Based on these findings, future studies should focus on evaluating fish responses to environmentally relevant concentrations of TBZ under natural conditions and assessing tissue residue levels with respect to public health implications.

## Figures and Tables

**Table 1:** Lethal concentration of tebuconazole for 24th, 48th, 72nd, and 96th h in rainbow trout.

Concentration (mg L^−1^)	Total Fish (n)	Number of Dead Fish (n)			
		24th h	48th h	72nd h	96th h
Control (0 mg L^−1^)	30	0	0	0	0
7.5 mg L^−1^	30	0	0	0	4
9.0 mg L^−1^	30	5	11	14	17
10.5 mg L^−1^	30	6	14	22	24
12.0 mg L^−1^	30	8	18	25	27
15.0 mg L^−1^	30	12	21	28	30
18.0 mg L^−1^	30	30	30	30	30
LC_10_ (mg L^−1^)		9.47 mg L^−1^	7.78 mg L^−1^	7.53 mg L^−1^	7.17 mg L^−1^
LC_50_ (mg L^−1^)		13.63 mg L^−1^	11.22 mg L^−1^	9.77 mg L^−1^	9.05 mg L^−1^
LC_90_ (mg L^−1^)		19.61 mg L^−1^	16.17 mg L^−1^	12.66 mg L^−1^	11.42 mg L^−1^

**Table 2:** Histopathological assessment tools for rainbow trout gills. Importance factor (1–3) is composed of the respective organ, the reaction pattern and the alteration. Score value is a Likert rating scale ranging from 0 to 6.

Tissue	Reaction Pattern	Alteration	Importance Factor	Score Value	Index
Gill	Regressive changes	Architectural and structural alterations	IF_1_ = 1	SV_1_ = 0–6	GI_RC_
	Progressive changes	Hypertrophy	IF_3_ = 1	SV_3_ = 0–6	GI_PC_
		Hyperplasia	IF_4_ = 2	SV_4_ = 0–6	

**Table 3:** Haematological parameters of rainbow trout in the control group (unexposed) and after exposure to the MTC and LC_50_-96 h of tebuconazole.

	Control	MTC	LC_50_	F	*p*
WBC	31.51 ± 3.0 ^a^	23.73 ± 3.9 ^b^	18.97 ± 2.5 ^b^	23.209	0.001
RBC	1.39 ± 0.1 ^a^	1.26 ± 0.2 ^ab^	1.06 ± 0.1 ^b^	7.134	0.007
HGB	12.25 ± 0.8 ^a^	10.8 ± 1.7 ^ab^	8.87 ± 0.6 ^b^	8.887	0.003
HCT	22.81 ± 2.6 ^a^	22.98 ± 3.0 ^a^	18.2 ± 1.3 ^b^	3.469	0.043
LYM	28.85 ± 2.6 ^a^	22.73 ± 3.5 ^b^	17.65 ± 2.3 ^c^	21.196	0.001
MCV	176.18 ± 2.2 ^ab^	181.37 ± 4.5 ^a^	171.45 ± 7.9 ^b^	5.319	0.018
MCH	87.9 ± 1.2 ^a^	85.35 ± 4.3 ^a^	83.4 ± 2.8 ^a^	0.731	0.498
MCHC	50.02 ± 1.2 ^a^	48.1 ± 1.9 ^b^	50.15 ± 2.2 ^a^	5.216	0.019

^abc^: Significant differences between groups for each reaction pattern depending on one-way ANOVA (*p* < 0.01).

**Table 4:** Severity of different histological alterations in gill tissues.

Tissues	Reaction Pattern	Symptoms	Severities		
			Control	MTC	LC_50_
Gill	Regressive changes	Architectural and structural alterations	+	++	++++
	Progressive changes	Hyperplasia	+	++	++++
		Hypertrophy	-	+	+++

(-): None, (+): mild, (++): moderate, (+++): severe, (++++): very severe.

**Table 5:** The reaction indices of histological alterations in control, MTC and LC_50_. RC: Regressive changes, PC: progressive changes and OI: organ index.

		RC	PC	OI
Gill	Control	1.12 ± 0.22 ^c^	3.58 ± 0.90 ^c^	4.70
	MTC	2.29 ± 0.44 ^b^	6.53 ± 0.69 ^b^	8.82
	LC_50_	5.32 ± 1.13 ^a^	15.44 ± 3.72 ^a^	20.76
	*F* values	91.259	75.461	

^abc^: Significant differences between groups for each reaction pattern depending on one-way ANOVA (*p* < 0.01).

## Data Availability

All datasets used during the current study are available from the corresponding author on reasonable request.
